# Supplementary material for: Pyruvate Dehydrogenase Kinase 1 inhibition mediated oxidative phosphorylation enhancement in cartilage promotes osteoarthritis progression
Source: BMC Musculoskelet Disord. 2023 Jul 20;24:597. doi: 10.1186/s12891-023-06585-6 (PMC10357736; doi:10.1186/s12891-023-06585-6)
Supplement: Supplementary file 1 — Additional file 1. [file 12891_2023_6585_MOESM1_ESM.zip › BP.Gsea.1653623667859/GOBP_GPI_ANCHOR_METABOLIC_PROCESS.html]

Details for gene set GOBP\_GPI\_ANCHOR\_METABOLIC\_PROCESS[GSEA]

|  || Dataset | 55457\_collapsed\_to\_symbols.cls.cls #OA\_versus\_normal.cls.cls #OA\_versus\_normal\_repos |
| Phenotype | cls.cls#OA\_versus\_normal\_repos |
| Upregulated in class | OA |
| GeneSet | GOBP\_GPI\_ANCHOR\_METABOLIC\_PROCESS |
| Enrichment Score (ES) | 0.57015777 |
| Normalized Enrichment Score (NES) | 1.9532629 |
| Nominal p-value | 0.002247191 |
| FDR q-value | 0.029062506 |
| FWER p-Value | 0.565 |
Table: GSEA Results Summary

  

Fig 1: Enrichment plot: GOBP\_GPI\_ANCHOR\_METABOLIC\_PROCESS      
 Profile of the Running ES Score & Positions of GeneSet Members on the Rank Ordered List

  

| SYMBOL | TITLE | RANK IN GENE LIST | RANK METRIC SCORE | RUNNING ES | CORE ENRICHMENT || 1 | GPAA1 | glycosylphosphatidylinositol anchor attachment 1 [Source:HGNC Symbol;Acc:HGNC:4446] | 44 | 1.006 | 0.1014 | Yes |
| 2 | PIGC | phosphatidylinositol glycan anchor biosynthesis class C [Source:HGNC Symbol;Acc:HGNC:8960] | 202 | 0.816 | 0.1744 | Yes |
| 3 | PIGP | phosphatidylinositol glycan anchor biosynthesis class P [Source:HGNC Symbol;Acc:HGNC:3046] | 367 | 0.718 | 0.2365 | Yes |
| 4 | PIGT | phosphatidylinositol glycan anchor biosynthesis class T [Source:HGNC Symbol;Acc:HGNC:14938] | 444 | 0.686 | 0.3021 | Yes |
| 5 | PIGO | phosphatidylinositol glycan anchor biosynthesis class O [Source:HGNC Symbol;Acc:HGNC:23215] | 462 | 0.675 | 0.3712 | Yes |
| 6 | PIGF | phosphatidylinositol glycan anchor biosynthesis class F [Source:HGNC Symbol;Acc:HGNC:8962] | 798 | 0.570 | 0.4046 | Yes |
| 7 | PIGV | phosphatidylinositol glycan anchor biosynthesis class V [Source:HGNC Symbol;Acc:HGNC:26031] | 1064 | 0.503 | 0.4365 | Yes |
| 8 | PGAP2 | post-GPI attachment to proteins 2 [Source:HGNC Symbol;Acc:HGNC:17893] | 1669 | 0.403 | 0.4316 | Yes |
| 9 | MPPE1 | metallophosphoesterase 1 [Source:HGNC Symbol;Acc:HGNC:15988] | 1675 | 0.402 | 0.4732 | Yes |
| 10 | PGAP3 | post-GPI attachment to proteins phospholipase 3 [Source:HGNC Symbol;Acc:HGNC:23719] | 2322 | 0.324 | 0.4567 | Yes |
| 11 | PIGN | phosphatidylinositol glycan anchor biosynthesis class N [Source:HGNC Symbol;Acc:HGNC:8967] | 2484 | 0.304 | 0.4760 | Yes |
| 12 | PIGH | phosphatidylinositol glycan anchor biosynthesis class H [Source:HGNC Symbol;Acc:HGNC:8964] | 2573 | 0.294 | 0.4998 | Yes |
| 13 | DPM3 | "dolichyl-phosphate mannosyltransferase subunit 3, regulatory [Source:HGNC Symbol;Acc:HGNC:3007]" | 2618 | 0.289 | 0.5265 | Yes |
| 14 | PIGB | phosphatidylinositol glycan anchor biosynthesis class B [Source:HGNC Symbol;Acc:HGNC:8959] | 2663 | 0.284 | 0.5527 | Yes |
| 15 | PIGK | phosphatidylinositol glycan anchor biosynthesis class K [Source:HGNC Symbol;Acc:HGNC:8965] | 2801 | 0.270 | 0.5702 | Yes |
| 16 | PIGQ | phosphatidylinositol glycan anchor biosynthesis class Q [Source:HGNC Symbol;Acc:HGNC:14135] | 4825 | 0.085 | 0.4219 | No |
| 17 | DPM2 | "dolichyl-phosphate mannosyltransferase subunit 2, regulatory [Source:HGNC Symbol;Acc:HGNC:3006]" | 4862 | 0.083 | 0.4277 | No |
| 18 | GPLD1 | glycosylphosphatidylinositol specific phospholipase D1 [Source:HGNC Symbol;Acc:HGNC:4459] | 5103 | 0.064 | 0.4158 | No |
| 19 | PIGG | phosphatidylinositol glycan anchor biosynthesis class G [Source:HGNC Symbol;Acc:HGNC:25985] | 6452 | -0.039 | 0.3151 | No |
| 20 | CWH43 | cell wall biogenesis 43 C-terminal homolog [Source:HGNC Symbol;Acc:HGNC:26133] | 6648 | -0.053 | 0.3055 | No |
| 21 | DPM1 | "dolichyl-phosphate mannosyltransferase subunit 1, catalytic [Source:HGNC Symbol;Acc:HGNC:3005]" | 7723 | -0.135 | 0.2362 | No |
| 22 | PIGZ | phosphatidylinositol glycan anchor biosynthesis class Z [Source:HGNC Symbol;Acc:HGNC:30596] | 9047 | -0.235 | 0.1580 | No |
| 23 | PGAP4 | post-GPI attachment to proteins GalNAc transferase 4 [Source:HGNC Symbol;Acc:HGNC:28180] | 9347 | -0.259 | 0.1618 | No |
| 24 | PGAP1 | post-GPI attachment to proteins inositol deacylase 1 [Source:HGNC Symbol;Acc:HGNC:25712] | 9358 | -0.260 | 0.1881 | No |
| 25 | PIGA | phosphatidylinositol glycan anchor biosynthesis class A [Source:HGNC Symbol;Acc:HGNC:8957] | 9571 | -0.280 | 0.2008 | No |
| 26 | PIGL | phosphatidylinositol glycan anchor biosynthesis class L [Source:HGNC Symbol;Acc:HGNC:8966] | 11817 | -0.553 | 0.0841 | No |
Table: GSEA details [plain text format]

  

Fig 2: GOBP\_GPI\_ANCHOR\_METABOLIC\_PROCESS      
 Blue-Pink O' Gram in the Space of the Analyzed GeneSet

  

Fig 3: GOBP\_GPI\_ANCHOR\_METABOLIC\_PROCESS: Random ES distribution      
 Gene set null distribution of ES for **GOBP\_GPI\_ANCHOR\_METABOLIC\_PROCESS**
